# High-intensity, but not moderate-intensity, exercise increases post-exercise rate of fat oxidation in type 2 diabetics

**Published:** 2016-05-21

**Authors:** Ricardo Yukio Asano, Marcelo Magalhães Sales, Rodrigo Alberto Vieira Browne, José Fernando Vila Nova de Moraes, Rafael da Costa Sotero, Gisela Arsa, Jonato Prestes, Johanna Lopez, John Eugene Lewis, Herbert Gustavo Simões

**Affiliations:** 1 *Faculty of Science and Letters, Municipal Foundation of Higher Education of Bragança Paulista, Bragança Paulista SP, Brazil*; 2 *Department of Physical Education, University of Mogi das Cruzes, Mogi das Cruzes SP, Brazil*; 3 *Department of Physical Education, State University of Goiás, Quirinópolis GO, Brazil*; 4 *Graduate Program in Physical Education, Federal University of Rio Grande do Norte, Natal RN, Brazil*; 5 *Graduate Program in Physical Education, Federal University of Vale do São Francisco, Petrolina PE, Brazil*; 6 *Department of Physical Education, Catholic University of Brasília, Brasília DF, Brazil*; 7 *Graduate Program in Physical Education, Federal University of Mato Grosso, Cuiabá MT, Brazil*; 8 *Graduate Program in Physical Education, Catholic University of Brasília, Brasília DF, Brazil*; 9 *Clinical Dietitian and Researcher, University of Miami Miller School of Medicine, Miami FL, USA*; 10 *Department of Psychiatry and Behavioral Sciences, University of Miami Miller School of Medicine, Miami FL, USA*

**Keywords:** metabolic disease, adipose tissue, oxidation, blood glucose, physical exercise, diabetes

## Abstract

**Background:**

Aerobic exercise is recommended for glycemic and weight control in type 2 diabetes (T2D), but exercise intensity that increase post-exercise fat oxidation has not been established yet. It is expected that high-intensity exercise induce higher absolute oxidations and rates of oxidation of CHO (during) and fat (after) in normoglycemic, but in hyperglycemic it is unclear.

**Aim:**

To compare the effects of exercise intensity on CHO and fat oxidation during and after exercise in individuals with T2D.

**Methods:**

Eleven persons with T2D, randomly underwent three experimental sessions 72 hours apart: 1) 20 minute of high-intensity exercise (120% of lactate threshold (LT) – 120%LT), 2) 20 minute of moderate-intensity exercise (80% of LT – 80%LT), and 3) 20 minute of control session (CON) – no exercise was performed and the individuals remained seated during the whole time. Percentages of CHO and fat contribution and CHO and fat oxidation rate (mg/min) were analyzed during and after sessions.

**Results:**

The rate of CHO oxidation during exercise was significantly higher during 120%LT in relation to 80%LT and CON (18.2 ± 5.6 *vs*. 9.5 ± 2.7 *vs*. 1.1 ± 0.4 mg∙min^−1^), the absolute rate of fat oxidation was significantly higher in 120%LT compared to 80%LT and CON during exercise (13.5 ± 3.3, 9.5 ± 2.2, and 0.7 ± 0.2 mg∙min^−1^, respectively, *p* < 0.05). During the post-exercise oxygen consumption recovery period, only the 120%LT had higher fat oxidation (94.5% *vs*. 68.1%, *p* < 0.05), when compared to CON. Both exercise sessions equally elicited a lowered glycaemia during the post-exercise period, but CHO oxidation was lower after 120%LT than CON (0.1 ± 0.2 *vs*. 0.9 ± 0.5 mg∙min^−1^, *p* < 0.05).

**Conclusions:**

Higher intensity elicited an elevated CHO oxidation rate during exercise and a higher percentage of fat utilization during the post-exercise recovery period compared to moderate-intensity exercise and control sessions.

**Relevance for patients:**

High-intensity aerobic exercise, even of short duration, may benefit individuals with T2D on the substrate oxidation related to the body fat. Exercise can be an important tool for the prevention and management of T2D due to its effects on carbohydrate and fat metabolism, reduction of body fat, and control of blood glucose.

## 1. Introduction

Type 2 diabetes (T2D) is a metabolic disease characterized mainly by insulin resistance resulting in hyperglycemia and disorders in carbohydrate, protein, and fat metabolism [1,2]. T2D is among the most prevalent non-transmissible diseases in Brazil, affecting more than 14 million individuals [3].

T2D can be managed pharmacologically or non- pharmacologically through lifestyle modifications that include dietary changes and proper exercise [1,2]. Exercise is the most important tool for the prevention and management of T2D due to its effects on carbohydrate and fat metabolism, reduction of body fat, and control of blood glucose [1,2]. The acute effects of each exercise session depend on its intensity and duration. These characteristics may determine the type of substrate used during and perhaps after exercise [4]. The uptake of blood glucose during exercise is important for the T2D patient to achieve and maintain normal blood glucose levels. Braun et al. [4] observed that during exercise the rate of disappearance of glucose and the percentage of plasma glucose used was not different between individuals with and without insulin resistance. Therefore, at least during exercise the ability to use carbohydrate seemed to be preserved in individuals with T2D [5]. On the other hand, the post-exercise recovery period is less studied regarding carbohydrate usage.

Today, moderate-intensity aerobic exercise, i.e., around the lactate threshold (LT), is usually recommended for blood glucose control in individuals with T2D [5]. However, high-intensity aerobic exercise could be beneficial for this population because it promotes the recruitment of more muscle fibers, with a higher increase in oxygen consumption, together with an oxygen deficit that needs to be restored after exercise is completed [6]. Thus, the so-called post-exercise oxygen consumption (EPOC) is necessary for several physiological mechanisms related to recovery of all body tissues. During EPOC, energy expenditure is increased, mostly in the form of fat oxidation [6]. For individuals with T2D, EPOC (and the possibility of increased post-exercise fat oxidation) may be beneficial to avoid excessive body fat accumulation, especially in the abdominal area, which is related both to insulin resistance and risk factors for cardiovascular complications [7,8].

Although moderate-intensity exercise can be beneficial for glycemic control, the optimal intensity of exercise to maximize post-exercise fat oxidation in individuals with T2D has not been established yet. With increasing exercise intensity, the body has a greater reliance on carbohydrate as a source of fuel, activating the catecholamine signaling pathway to increase hepatic glucose production and transient hyperglycemia that would last 1-2 hours post-exercise [9]. Therefore, performing maximum intensity exercise can promote risks inherent to the disease itself, which precludes applying this prescription to the referred population. On the other hand, high-intensity exercise has been shown to be more effective for post-exercise blood pressure control [9]. However, the effect of exercise intensity on the amount of fat and carbohydrate oxidation during and after exercise needs clarification to optimize exercise prescription for T2D. Thus, this study tested the hypothesis that high-intensity aerobic exercise (i.e., above LT) would maximize carbohydrate oxidation during exercise and potentiate fat oxidation during the post-exercise recovery period in individuals with T2D.

## 2. Materials and Methods

### 2.1. Sample

After approval from the Research and Ethics Committee of the Catholic University of Brasília (protocol number 167/2011) and signing of the informed consent form (Resolution 466/2012 of the Brazilian National Health Counsel and provisions of the Declaration of Helsinki), 11 individuals (5 men and 6 women) clinically diagnosed with T2D from the city of Brasília-DF participated in the study. Participants were nonsmokers with an average age of 62.1 ± 9.0 years, body mass of 74.7 ± 12.2 kg, body mass index of 28.8 ± 4.6 kg∙m^−2^, fasting blood glucose of 154.7 ± 56.8 md∙dL^−1^, systolic blood pressure (SBP) of 129.5 ± 10.1 mmHg, diastolic blood pressure (DBP) of 73.1 ± 10.3 mmHg, and VO_2_ peak of 21.4 ± 4.5 md∙kg^−1^∙ min^−1^. The confirmation of diabetes diagnosis was conducted through a medical evaluation and a fasting glucose test. All individuals were under medical and nutritional treatment, using one oral hypoglycemic medication (Sulfonylureas, Metformin, Glibenclamide + Metformin, Glimepiride, Pioglitazone Hydrochloride) and/or food intake control. Furthermore, four were under use of antidiuretic (Chlorpropamide).

The exclusion criteria included a diagnosis of peripheral autonomic neuropathy, for which the following aspects were considered: resting heart rate (HR) higher than 90 beats per minute, incapacity of reaching 85% of the predicted maximum HR for age during the maximal incremental exercise test, reducing less than 12 beats per minute during the first minute after finishing the incremental test, and abnormal HR variability [10]. The participants could not have ulcers characteristic of diabetic foot or any other orthopedic impairment that could preclude performing exercise. Furthermore, the individuals could not have been on insulin or any other medicine that could interfere with the outcome variables to be evaluated.

### 2.2. General procedures

All experimental sessions were performed in the Physical Evaluation and Training Laboratory (LAFIT) at the Catholic University of Brasília. All medication was washed-out for 24h prior to the initial screening visit and the three subsequent sessions. The individuals were also asked to avoid physical exercises and alcoholic or caffeinated drinks for 24h prior to each visit to the laboratory. Two hours after ingestion of a standard moderate glycemic index (GI = 73.9) breakfast that provided a total of 315.9 kcal, with 53 g (61.7% - 212 kcal) of carbohydrate, 4.6 g (5.8% - 18.3 kcal) of protein, and 9.5 g (27.1% -85.6 kcal) of fat. The participants also underwent a clinical evaluation including a resting electrocardiogram (ELITE, Micromed®), blood pressure (BP) measurements (BP 3AC1-1 Microlife Co.), anthropometry, and a maximal incremental exercise test (MIT) on a cycle ergometer. During the MIT, HR, BP, rating of perceived exertion (RPE), ventilation, and blood lactate were continuously monitored.

### 2.3. Anthropometric measurements

Body mass index (BMI) was calculated considering the ratio of body mass (Toledo 2096 PP) in kilograms and height in meters (stadiometer SECA® 214, USA) raised to the second power (kg∙m^−2^). The percentage body fat was estimated from the technical skinfold, wherein the body density was calculated using the seven folds protocol suggested by Jackson and Pollock [11], collected at each point in rotational sequence, the right side of the body, and logged a mean value of three measurements. The measurements were performed by a single examiner, using a skinfold caliper (Lange, Cambridge Scientific Instruments, Cambridge, Maryland, USA). After calculating the body density, it was converted into fat percentage using the equation proposed by Siri [12].

### 2.4. Maximal incremental test

Maximal heart rate, lactate threshold, and VO_2_ peak were determined during the MIT. After collecting medical history and anthropometric variables, participants performed the MIT on a cycle ergometer (Lode Excalibur, Netherlands) with an initial load of 15 W, followed by a 15 W increase at each 3 minute stage at a speed of 60 revolutions per minute until volitional exhaustion. During MIT, the electrocardiogram of the volunteers was monitored by a cardiologist. The following criteria were used to determine whether participants achieved maximal effort: respiratory-exchange ratio (RER) ≥ 1.1, HR > 90% maximum predicted by age and RPE > 17 [13].

Before exercise and during the last 20 seconds of each stage, 25 µL of capillary blood was collected from the earlobe using disposable lancets and calibrated and heparinized glass capillaries. The blood samples were deposited in microtubes (Eppendorf) containing 50 µL of sodium fluoride (NaF) at 1% for analysis of lactate concentration, using the electro-enzymatic method (Yellow Springs 2.700 STAT, OH, USA).

Gas exchange during the MIT was obtained through facemask of the Metalyzer 3B Gas Analyzer (Cortex Boiphysik, Germany) previously calibrated with a 3 L syringe (calibration flux) and a mixed pattern of gas containing 4.9% of CO_2_ and 17% of O_2_ (calibration gas). Ventilation, oxygen uptake (VO_2_), and carbon dioxide production (VCO_2_) were registered during the whole procedure, with the last 20 seconds of every 3 minute stage being analyzed. In addition, BP was collected through the auscultatory method using a sphygmomanometer and a stethoscope (Tycos Hospital Instruments, São Paulo, Brazil) during the last 60 seconds of each 3 minute stage. All equipment was calibrated according to manufacturer’s instructions.

### 2.5. Lactate threshold determination

To determine the LT, lactate concentration kinetics were examined during the MIT stages. Based on previous reports, LT was determined at the intensity of exercise when the lactate concentration curve increased exponentially, and blood glucose minimum, ventilatory threshold, relationship VCO_2_/VO_2_ were used to confirm lactate threshold workload [14,15].

### 2.6. Experimental sessions (80% and 120% of lactate threshold load)

In these sessions, the volunteers performed 20 minutes of aerobic exercise on a cycle ergometer (Lode Excalibur, Netherlands) with a relative intensity of 80% (80%LT – moderate intensity) and 120% (120%LT – high intensity) of the LT load that was previously determined during the MIT. The order of sessions (120%LT, 80%LT, and control session (CON)) was randomized, and the minimum and maximum intervals between sessions were 72 and 120 hours, respectively. In order to try to isolate the effect of exercise on metabolic activity, individuals were prevented from receiving any visual stimulus in the recovery period. Therefore, no activity of reading, for example, was allowed during this period. Thus, individuals were instructed to remain seated until the 45^th^ minute.

### 2.7. Control session

The CON followed every procedure applied in 80%LT and 120%LT. However, the participants remained seated in a resting position without performing exercise.

### 2.8. Measurements performed in the experimental sessions

**Figure 1.**
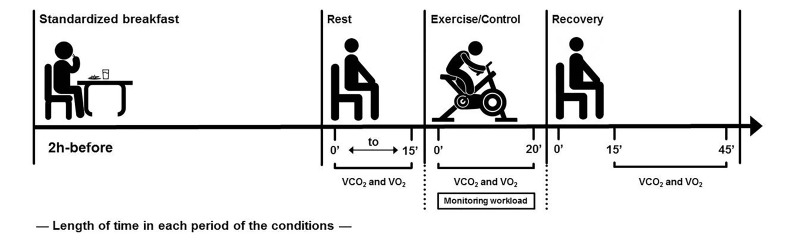
Summary of experimental design.

The experimental design of the exercise and CON is presented in Figure 1. In all sessions, expired gases were measured during 15 minutes of pre-intervention rest, at each 5 minutes of intervention (20 minutes), and at the 15^th^ and 45^th^ minute of post-intervention recovery period. Blood lactate samples (25 µL), % maximal HR, rate-pressure product (SBP (mmHg) × HR (bpm)) and RPE were monitored during the sessions every 5 minutes (minutes 5^th^, 10^th^, 15^th^ and 20^th^) and the average was considered.

### 2.9. Ventilation measurements to determine respiratory exchange ratio and substrate oxidation

VCO_2_ and VO_2_ were measured continuously during exercise and the post-exercise recovery period and allowed for the calculation of the RER by dividing the variables (VCO_2_/VO_2_) [16]. RER was used to calculate the percentage of carbohydrate and fat used as energy substrate. Exclusive fat oxidation was determined when the RER value was 0.70, while exclusive carbohydrate oxidation occurred at an RER value of 1.0 [16]. In addition, the carbohydrate oxidation rate was estimated by the following equation as suggested by Braun et al. [4]:

Carbohydrate oxidation rate (mg∙min^−1^) = [(% of carbohydrate oxidation/100) × (VO_2_ L∙min^−1^) × (5.05 kcal∙L^−1^)/(4.0 g∙kcal^−1^)]

Fat oxidation rate (mg∙min^−1^) = [(% of fat oxidation/100) × (VO_2_ L∙min^−1^) × (5.05 kcal∙L^−1^)/9.0 g∙kcal^−1^]

### 2.10. Statistical analysis

The minimum sample size calculated by G-power Software 3.0.10 with statistical power of 80% and an alpha of 0.05 was 11. The normality and homogeneity of variance were tested using the Shapiro-Wilk and Levene tests, respectively. Data were analyzed using means ± standard deviations. To compare the values of RPE, a paired t-test was performed. To compare the mean values of VO_2_, blood lactate, % maximal HR, rate-pressure product, % oxidation, and rate of fat and carbohydrate oxidation between conditions, a one-way ANOVA was performed with Bonferroni *post-hoc* comparisons. To compare VO_2_, RER and blood lactate within and between conditions, a repeated-measure ANOVA was performed with Bonferroni *post-hoc* comparisons. The hypothesis of sphericity was verified by Mauchly test and, when violated, the degrees of freedom are corrected by the Greenhouse-Geisser estimates. All statistical procedures were performed using SPSS 15 (IBM, Inc, Chicago, IL, USA). The level of statistical significance was α = 0.05.

## 3. Results

The main characteristics of the participants are 62.1 ± 9.0 years old, body mass 74.8 ± 12.2 kg, stature 1.61 ± 0.1 m, BMI 28.8 ± 4.6 kg∙m^–2^, waist circumference 90.3 ± 10.0 cm and fasting glucose 154.7 ± 56.8 mg∙dL^−1^. The mean fasting glucose values demonstrate a hyperglycemic condition for the sample, and the VO_2_peak reveals low physical fitness levels of the participants.

The description of effort of the experimental conditions (80%LT, 120%LT, and CON) is presented in Table 1. VO_2_ was significantly higher in 120%LT compared to 80%LT and CON and when comparing 80%LT to CON. Blood lactate was higher only in 120%LT compared to CON. Rate-pressure product was higher in 120%LT and 80%LT compared to CON. RPE was higher in 120%LT compared to 80%LT, validating that both exercise intensities were different.

**Figure 2.**
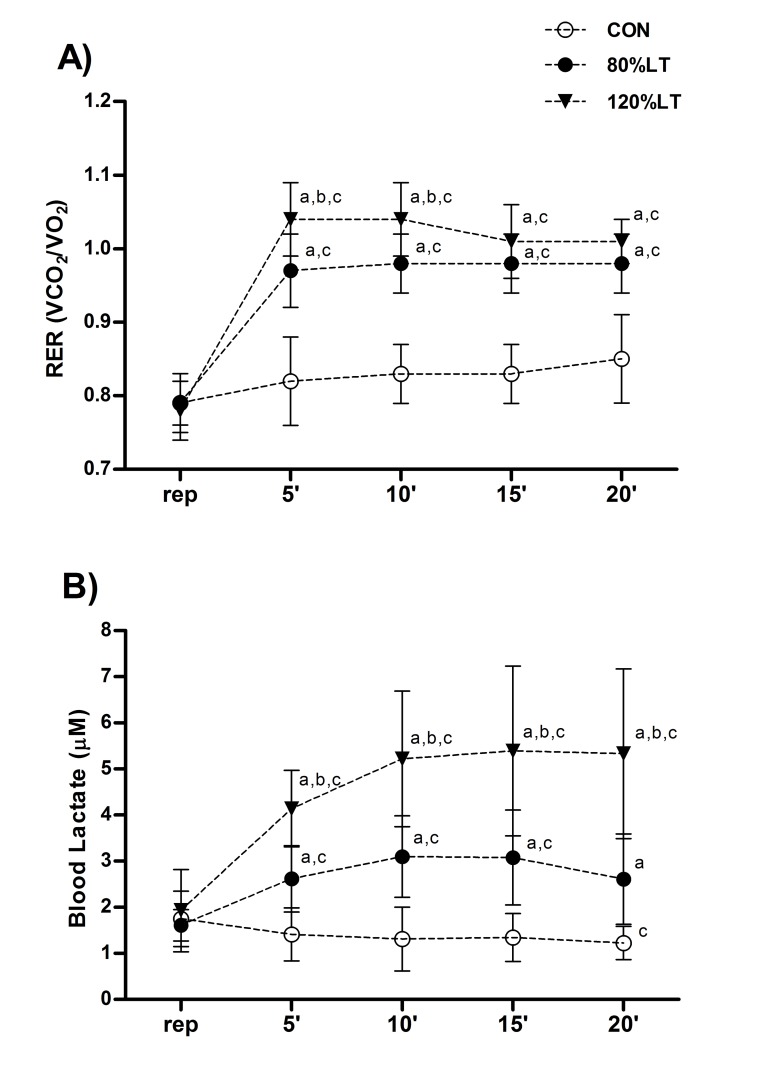
Respiratory-exchange ratio (VCO_2_ / VO_2_) (A) and blood lactate (B) at each 5 min during intervention (5’ – 20’) for the exercise and control conditions. Values are mean (± SD). CON, control session; 120%LT, exercise at 120% of lactate threshold; 80%LT, exercise at 80% of lactate threshold. ^a^ *p* < 0.05 at the same time to CON; ^b^ *p* < 0.05 at the same time to 80%LT; ^c^ *p* < 0.05 to rest at the same session.

In addition, Figure 2 shows the RER and blood lactate at each 5 min during exercise and control conditions. The RER of 80%LT and 120%LT conditions was higher than CON, but 120%LT was even greater than 80%LT in minutes 5^th^ and 10^th^ (Figure 2A). Similarly, blood lactate of 80%LT and 120%LT conditions was higher than CON, but that of 120%LT was even greater than of 80%LT at all times (5' – 20') (Figure 2B).

**Table 1.** Mean values for VO_2_, blood lactate, % maximal HR, rate-pressure product (SBP × HR), and RPE during the exercise and control conditions (CON, 80%LT, and 120%LT).

	CON	80%LT	120%LT
VO_2_ (mL∙kg^−1^∙min^−1^)	2.9 ± 0.4	12.9 ± 2.6^a^	17.6 ± 3.0^a^,^b^
Blood Lactate (μM)	1.3 ± 0.5	2.8 ± 0.9	5.2 ± 1.5^a^
HRmax (%)	46.5 ± 6.9	77.5 ± 7.1	92.6 ± 11.1
Rate-pressure product	7632.1 ± 1184.3	15803.3 ± 1989.7^a^	17898.0 ± 2723.1^a^
RPE (score)		11.0 ± 1.3	13.0 ± 0.6^b^

Values are mean ± SD. CON, control session; HRmax, maximal heart rate; RPE, rating of perceived exertion; SBP, systolic blood pressure; VO_2_, oxygen consumption; 120%LT, exercise condition at 120% of lactate threshold; 80%LT, exercise condition at 80% of lactate threshold.

a *p* ≤ 0.05 to CON;

b *p* ≤ 0.05 to 80%LT.

**Figure 3.**
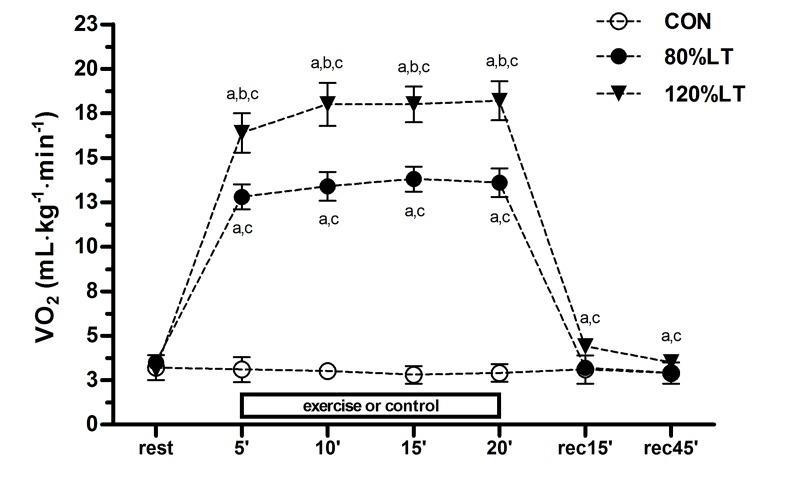
Oxygen consumption (VO_2_) at each 5 min of intervention (5’ – 20’) and during the recovery period (rec15’ – rec45’) for the exercise and control conditions. Values are mean (± SD). CON, control session; 120%LT, exercise at 120% of lactate threshold; 80%LT, exercise at 80% of lactate threshold. ^a^ *p* < 0.05 at the same time to CON; ^b^ *p* < 0.05 at the same time to 80%LT; ^c^ *p* < 0.05 to rest at the same session.

**Figure 4.**
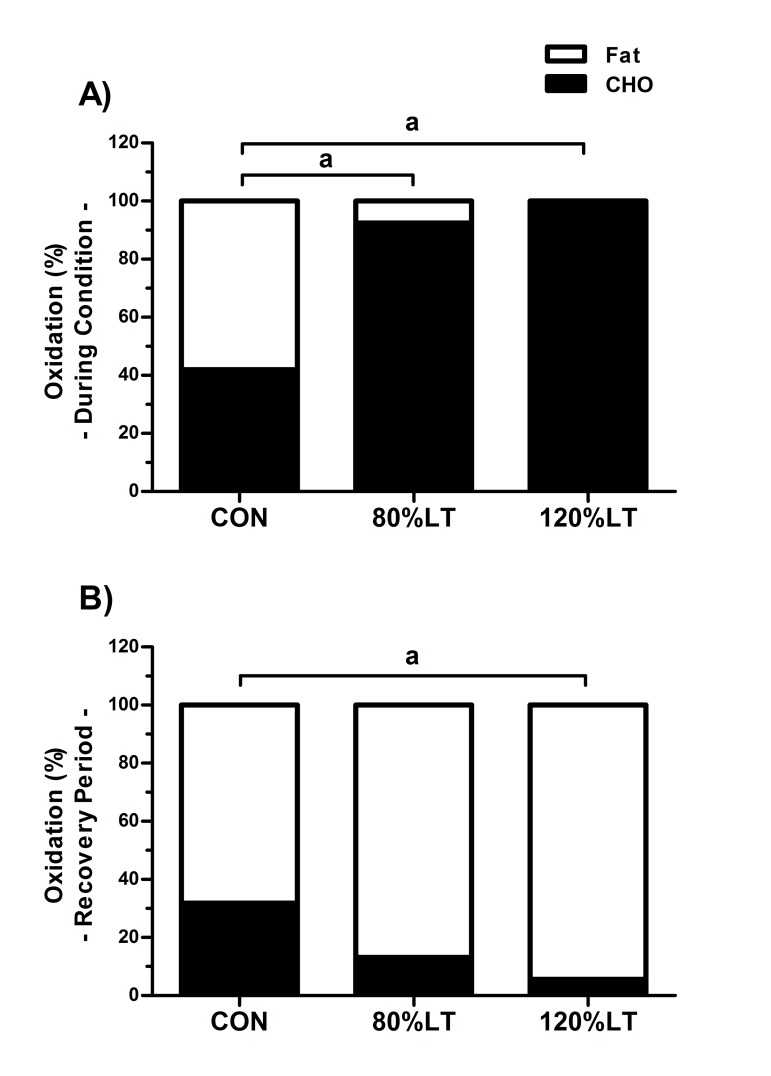
Mean percentage of carbohydrate (CHO) and fat oxidation during exercise and control conditions (A), and at the recovery period (B). CON – control session. 80%LT – exercise condition at 80% of lactate threshold. 120%LT – exercise condition at 120% of lactate threshold. ^a^ *p* ≤ 0.05 *vs*. CON.

The mean VO_2_ values for 120%LT were significantly higher than 80%LT and CON for the exercise and post-exercise recovery periods (Figure 3), and EPOC occurred only after 120%LT (p < 0.05). Both exercise groups elicited glycemic reductions compared to values observed at the 45^th^ minute of post-exercise recovery in each experimental condition. For participants under treatment with an oral hypoglycemic, blood glucose dropped from 121.2 ± 14.7 to 90.7 ± 9.1 md∙dL^−1^ in the 120%LT condition, from 127.2 ± 20.2 to 102.1 ± 18.5 md∙dL^−1^ in the 80%LT group (*p* < 0.05), and no change for the CON group. These results corroborate the findings that the percentage of carbohydrate used as energy substrate (120%LT = 99.1% and 80%LT = 92.4%) during exercise was significantly higher compared to CON (41.8%) (Figure 4), substantiating the role of exercise for carbohydrate oxidation and blood glucose control in individuals with T2D.

**Figure 5.**
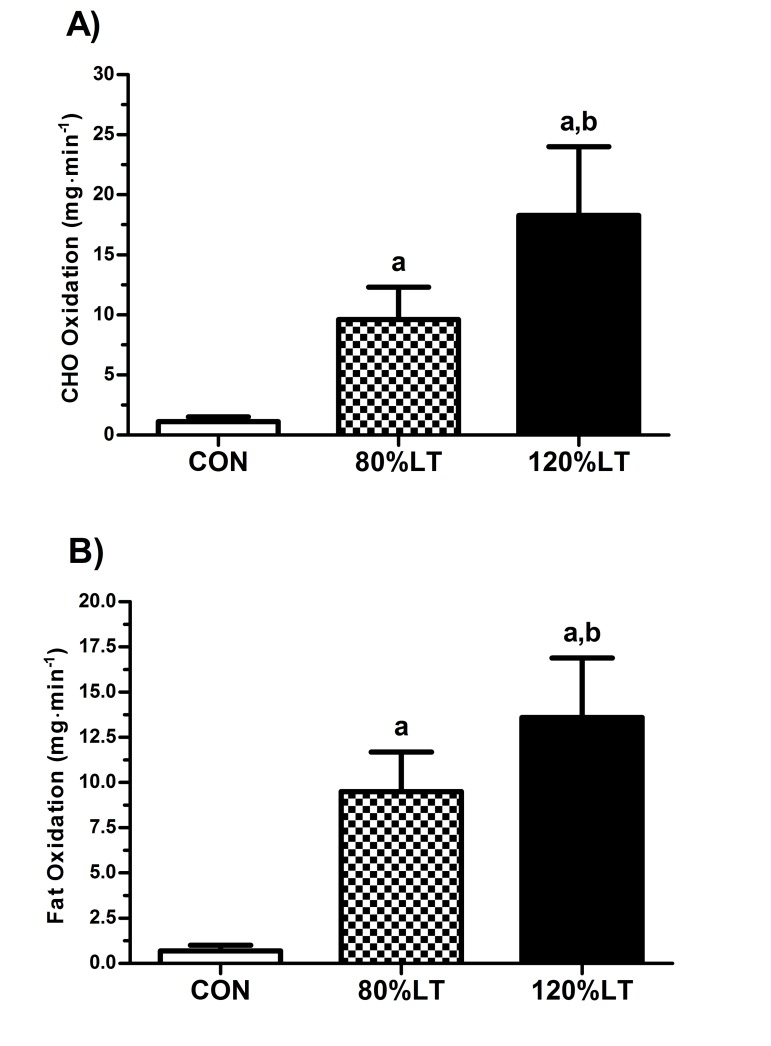
Rate of carbohydrate (A) and fat (B) oxidation during conditions. Values are mean (± SD). CHO, carbohydrate; CON, control session; 120%LT, exercise at 120% of lactate threshold; 80%LT, exercise at 80% of lactate threshold. ^a^ *p* ≤ 0.05 *vs*. CON; ^b^ *p* ≤0.05 *vs*. 80%LT.

Besides enabling EPOC (Figure 3), the 120%LT group also showed a higher percentage of fat oxidation (94.5%) during post-exercise recovery (*p* ≤ 0.05) compared to CON (68.1%) (Figure 4B). The rate of carbohydrate oxidation during exercise was significantly higher in 120%LT (18.2 ± 5.6 mg∙min^−1^) compared to 80%LT and CON (9.5 ± 2.7 and 1.1 ± 0.4 mg∙min^−1^, respectively, *p* < 0.05) (Figure 5A). Similarly, the absolute rate of fat oxidation during exercise was significantly higher in 120%LT compared to 80%LT and CON (13.5 ± 3.3, 9.5 ± 2.2, and 0.7 ± 0.2 mg∙min^−1^, respectively, *p* < 0.05) (Figure 5B).

## 4. Discussion

T2D promotes several metabolic alterations during the use of substrates [8,17]. The percentage of energy derived from the oxidation of carbohydrate and fat in individuals with T2D is different from those without T2D [5,18]. Impaired glucose uptake leading to hyperglycemia is related to a higher content of intramuscular fat, which is associated with insulin resistance [6,19].

The present study analyzed the role of exercise intensity on parameters related to carbohydrate and fat oxidation during and after exercise in individuals with T2D. The main findings are that aerobic exercise performed at 120% of LT is more effective in increasing carbohydrate oxidation during exercise, produces glycemic control during and after exercise, and causes an increased percentage of fat oxidation during the post-exercise recovery compared to aerobic exercise performed at 80%LT and the control condition. These results may be related to EPOC that was observed only after high-intensity (120%LT) exercise training.

Higher intensity exercise was associated with more: neuromuscular activity, muscle mass involved, and metabolic demand during and after exercise. [9]. These adaptations may be related to a larger activity of endocrine and metabolic systems, together with elevated cardiovascular and respiratory responses leading to EPOC [7,8]. Thus, results indicated that the highintensity exercise elicit glycemic control and fat oxidation during post-exercise period by eliciting a higher metabolic demand. These effects are important for individuals with T2D since they may be related to blood glucose uptake and utilization by skeletal muscle, eliciting glycemic control and increased stored fat utilization, thus helping to reduce body fat [7] and in turn improving insulin sensitivity [2].

These results should be analyzed with caution for clinical purposes. Exercise must be prescribed on an individual basis, as high intensities should be avoided for some (if not most) persons with T2D. However, it is important to point out that the high-intensity exercise in the present study (120%LT) was actually submaximal. In such 20 minute exercise sessions, the VO_2_ and HR of the participants reached about 83% and 92% of their maximal values, respectively, and an RPE of 13. Additionally, instead of using 120% of LT, intensities around LT (e.g., between 90% and 110%LT) could be applied intermittently that could also be effective and less stressful for persons with T2D.

The findings are consistent with previous studies that demonstrated the importance of high-intensity aerobic exercise for carbohydrate and fat utilization during and after exercise in individuals with T2D. Lima et al. [20] investigated the effect of two different exercise intensities on a cycle ergometer (MIT and 90%LT) in nine individuals with T2D. Maximal exercise and 90%LT promoted high percentage of carbohydrate utilization than the rest condition, demonstrating that carbohydrate utilization during exercise is not impaired in T2D individuals. In other words, the glucose uptake mechanism independent of insulin appears to remain preserved in this population. However, only MIT exercise elicited increased fat utilization and oxidation after exercise (recovery period) in T2D compared to pre-exercise. Thus, for the present study we investigated 120% of LT that was high intensity, but not maximal. In another study from our group, Cunha et al. [21] investigated the effects of fat oxidation in 9 individuals with T2D and 11 non-diabetics after performing MIT and moderate intensity exercise (90%LT) and observed a significant increase in fat oxidation after MIT in the group with T2D. On the other hand, the non-diabetic group increased fat oxidation after both exercise intensities. These findings, along with ours, suggest that individuals with T2D benefit more from fat oxidation after exercise when higher-intensity exercise is performed, probably as a consequence of increased EPOC [22] that was observed in the present study only for the 120%LT intensity.

High intensity exercise can be extremely beneficial not only for improving body composition, but also because an increased oxidation of fat in the post-exercise recovery period can reduce intramuscular fat, reduce the formation of long-chain acylCoA in the interior of the muscle cell, inhibiting the formation of diacylglycerol and ceramides, and thus favoring the uptake and oxidation of glucose in the muscle cell [2]. Therefore, optimal exercise intensities need to be established to enhance fat oxidation during recovery in individuals with T2D, maximize insulin sensitivity, and decrease body fat percentage. Thus, the significance of exercise intensity, even a submaximal one, was validated in the present study.

Another benefit of aerobic exercise performed at higher intensity is the increased use of carbohydrates as an energy substrate. Further investigation is warranted on the effect this could have on transient hyperglycemia and how this can affect individuals with T2D [23,24].

However, the present study is not without limitations. Although indirect calorimetry is the gold standard for estimating energy expenditure, its results can be affected by the high production of non-metabolized CO_2_, which induces an increase in ventilatory responses [12]. This eliminates the excess CO_2_ and results in a change of RER toward lower values, suggesting a higher oxidation of fat. However, it is possible that respiratory alkalosis also may have contributed to the decreased post-exercise RER [25,26]. Thus, methods other than RER should be used in future studies to corroborate the results of the present study.

## 5. Conclusions

The results of the present study suggest that performing aerobic exercise above LT promotes an increase in carbohydrate oxidation during exercise and increases post-exercise fat oxidation in individuals with T2D. These findings are important for glycemic and body weight control in individuals with T2D. But, caution is warranted when applying these results, since the participants of the present study are individuals with controlled T2D with few or no associated complications. In addition, the exercise sessions performed in the present study took place in a highly controlled environment under monitoring by a cardiologist given that T2D patients usually have cardiovascular complications.
